# Operative Treatment of Tarlov Cysts - Outcomes and Predictors of Improvement after Surgery: A Series of 97 Consecutive Patients and a Systematic Review of Literature

**DOI:** 10.1177/21925682231221538

**Published:** 2023-12-09

**Authors:** Delshad Abdi, Jukka Huttunen, Ville Leinonen, Sakari Savolainen, Nils Danner

**Affiliations:** 1 Institute of Clinical Medicine, 101232 University of Eastern Finland, Kuopio, Finland; 2 Neurocenter, Neurosurgery, 60650 Kuopio University Hospital, Kuopio, Finland

**Keywords:** tarlov cyst, perineural cyst, sacrum, operative treatment, surgery, treatment outcome

## Abstract

**Study Design:**

A register-based retrospective series and a systematic review of literature

**Objectives:**

Tarlov cysts are meningeal cysts typically found in the sacral region. They have a dualistic nature ranging from an incidental finding to a symptomatic pathology. There are no established treatment protocols and predictors of operative outcome. Therefore, we aimed to study the outcome of surgical treatment for Tarlov cysts and to characterize patient-, and treatment-related factors predicting outcomes.

**Methods:**

A systematic review of previous literature was performed and a retrospective cohort of all patients operated on for Tarlov cysts at BLINDED between 1995 and 2020 was collected. Patient records were evaluated along with radiological images.

**Results:**

Ninety-seven consecutive patients were identified with follow-up data available for 96. Improvement of symptoms after surgery was observed in 76.0% of patients (excellent or good patient-reported outcome) and the complication rate was 17.5%. Sacral or lower back pain as a preoperative symptom was associated with improvement after surgery (*P* = .007), whereas previous lower back surgery was more common in patients who did not benefit from surgery (*P* = .034). No independent predictors of outcome were identified in a regression analysis.

**Conclusions:**

This is the second-largest study on the treatment of Tarlov cysts ever published. Operative treatment in a selected patient population will likely produce improvement in the symptoms when balanced with the complication rate and profile of surgery. Preoperative lower back or sacral pain is a potential indicator for improvement after surgery.

## Introduction

Tarlov cysts are cerebrospinal fluid-filled (CSF) enlargements, which arise between the perineurium and endoneurium of the nerve root sheath and appear at the junction of a posterior nerve root and dorsal root ganglion.^1–3^ They were originally reported in 1938 by Isadore M. Tarlov^4^ and are typically found in the sacral region but can occur at any level of the spine.^1–5^ Tarlov cysts, also named perineural cysts, are defined as Type II spinal meningeal cysts with nerve fibers inside the cyst or nerve tissue in the walls of the cyst.^1^ The precise etiology of Tarlov cysts remains undetermined, but cyst enlargement and growth are thought to be the consequence of valve-like micro communication between the cyst and subarachnoid space.^2^

Tarlov cysts are often incidental findings in magnetic resonance imaging (MRI) or computed tomography (CT).^2^ The global incidence of Tarlov cysts has been evaluated in a meta-analysis of radiological studies, and the pooled prevalence was estimated to be 4.2% with a higher prevalence in women.^5^ One-fifth of the cysts are regarded as symptomatic,^3,5^ typically causing lower back pain, sensorimotor disturbances of the lower limbs as well as bladder, bowel, or sexual dysfunctions.^2–4,6^ Regardless of the considerable prevalence of symptomatic Tarlov cysts, literature on their treatment is strikingly scarce. A wide array of treatment protocols for symptomatic cysts has been suggested ranging from percutaneous aspiration techniques to microsurgery with variable success rates and small patient populations.^2,3,6–16^

Since Tarlov cysts are seen to have a dualistic nature ranging from an incidental finding to a symptomatic pathology, there are no established treatment protocols and predictors of outcome. Therefore, we performed a systematic literature review on the treatment of Tarlov cysts. Furthermore, we conducted a retrospective study of all patients operated on for Tarlov cysts at BLINDED University Hospital (BLINDED) between 1995 and 2020. The aim was to describe outcomes of the applied surgical practice and to characterize patient-, and treatment-related factors predicting outcomes.

## Material and Methods

### Literature Review

**Figure 1.**
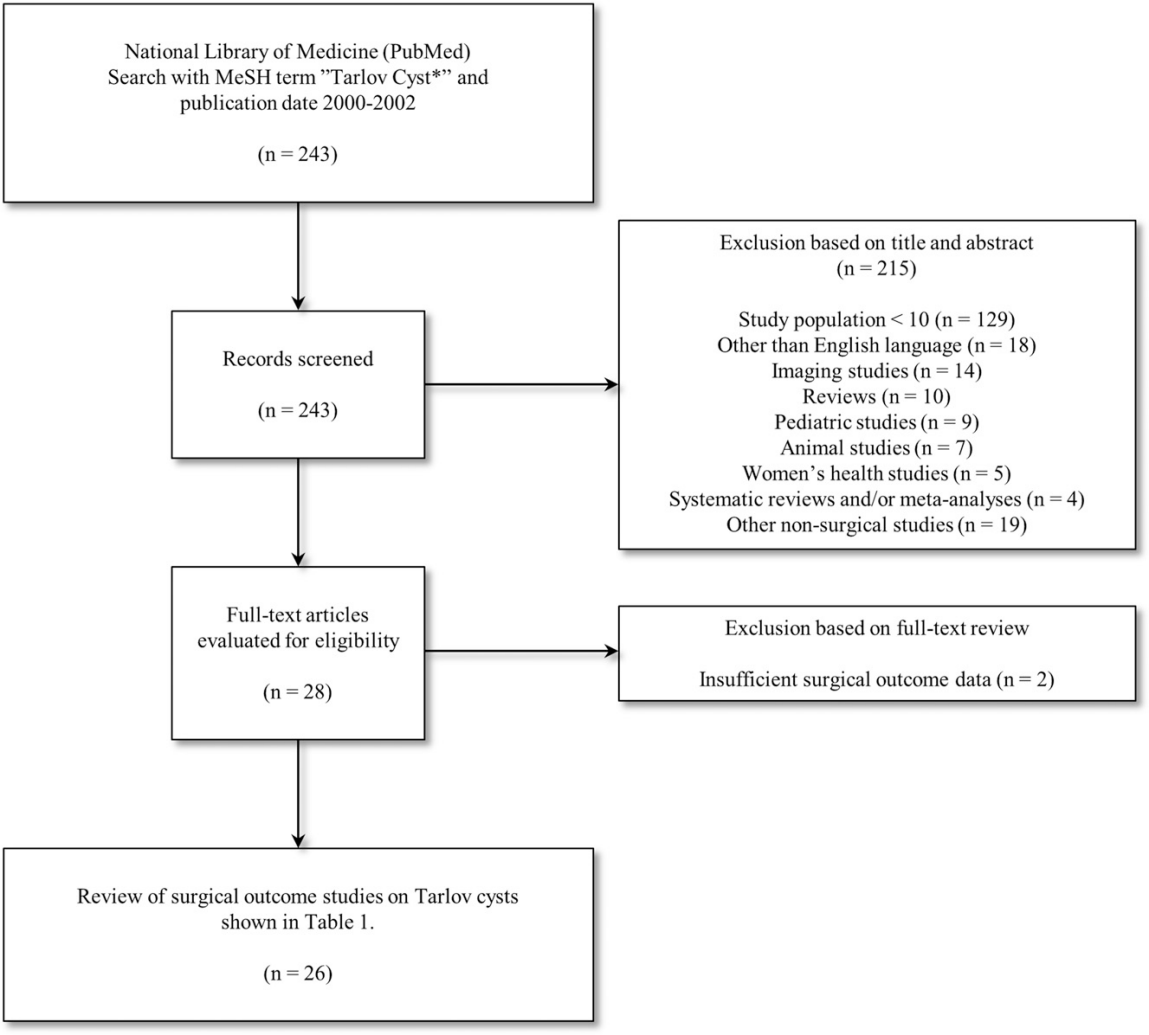
Flow chart of the systematic literature review.

The PubMed database was searched with the MeSH term ‘Tarlov cyst*’. Titles and, if required, abstracts were screened for further evaluation. The inclusion criteria were: (1) human studies, (2) original articles, (3) number of subjects 10 or more, (4) publication year 2000 or later, (5) English language. The flowchart of the search strategy shown in Figure 1.

### Case Selection

This retrospective study was conducted at BLINDED University Hospital (BLINDED). BLINDED neurosurgery is a tertiary center that provides surgical treatment of Tarlov cysts in the catchment area of BLINDED. Patients were identified from BLINDED hospital registers from 1995 to 2020. The operation registries were reviewed to identify all patients in digital or manual forms depending on the date of surgery. Clinical data were gathered from digital and manual patient files and imaging data from radiological archives (picture archiving and communication system, PennsylvaniaCS) as well as film images. The study was register-based, and therefore no informed consent was required. The research committee of BLINDED approved the study (approval no: 5772670). Data were processed in accordance with the requirements and guidelines of BLINDED.

### Symptom Categorization

In order to comprehensively characterize the symptomatology related to Tarlov cysts, pre-operative symptoms were recorded as described in patient reports. The symptoms were categorized by type and localization into 4 main groups: motor, sensory, bladder, and bowel. Sensory symptoms were further divided into pain and numbness/dysesthesia. The localization of symptoms was categorized as local (pelvis, lower back/sacral) or radicular (lower limb).

### Radiological Analysis

The number (single/multiple) and size of the cyst(s) were reviewed. The mean volume of the cyst(s) and the largest maximum diameter of each culprit cyst were reported. The volume of culprit cyst(s) was calculated using the ellipsoid volume formula (volume = 4/3 × π × a × b × c, π = 3.14, with a, b, and c representing the radiuses of the three axes).

### Outcome and Follow-Up

The postoperative follow-up was performed with outpatient appointments and/or telephone interviews. The length of the follow-up was determined from the operation to the last recorded patient contact. The patient-reported outcomes were categorized by the modified MacNab criteria,^17^ which divide outcomes into 4 categories: excellent, good, fair, and poor. For statistical analyses, the outcome was further dichotomized as “improvement” (excellent + good) and “no improvement” (fair + poor).

### Bias

To avoid observation bias, the surgeon involved in surgery did not participate in the clinical data collection or analysis.

### Statistical Analysis

Statistical analyses were performed using SPSS (version 27, IBM Corp.). Categorical variables were cross-tabulated and analyzed by the chi-square test or Fisher’s exact test. Continuous data were analyzed by the *t* test and the Mann-Whitney U. The limit of statistical significance was set to *P* < .05. Furthermore, a binary logistic regression analysis was performed to find possible independent predictors of outcome. Parameters used in the analysis were: age, sex, prior lower back surgery, lower back or sacral pain, reoperation, complication, and cyst number (single vs multiple). Radiological parameters and body mass index (BMI) were not included in the regression analysis due to missing data.

## Results

The flow chart of the systematic literature review is presented in Figure 1. A total of 243 abstracts were screened with the pre-defined inclusion and exclusion criteria. Of these, 28 studies were selected for full-text review. Following the full-text review, 2 studies were excluded due to insufficient surgical outcome data. The final analysis included a total of 26 studies, comprising 22 surgical studies and 4 percutaneous studies. Two patient populations were identified as duplicates, both reported twice in separate articles.^15,18–20^ The outcome was categorized as good when the outcome of the study was reported as excellent or good according to the modified MacNab criteria.^17^ All complications, symptomatic recurrences and reoperations were recorded. The results of the literature review are presented in Table 1.

**Table 1.** Literature Review of Treatment Outcomes for Tarlov Cysts.

Author and year	Number of patients	Mean age	Female patients	Multiple cysts	Complication rate	Reoperation rate	Good outcome	Symptomatic recurrence	Mean follow-up (months)
Surgical									
Burke et al, 2016^21^	23	49.3	83%	NA	21.7%	21.7%	70.0%	NA	14.4
Cantore et al, 2013^13^	19	45.4	74%	53%	0%	0%	84.2%	NA	122.6
Caspar et al, 2003^10^	15	45.0	67%	7%	0%	0%	86.7%	0%	60.0
Chu et al, 2022^4^	265	44.1	34%	34%	13.6%	NA	80.7%	.9%	44.7
Elsawaf et al, 2016^11^	15	31.0	67%	NA	13.3%	0%	100.0%	0%	54.0
Fletcher-Sandersjöö et al, 2019^2^	17	53.0	72%	72%	0%	0%	94.1%	0%	62.0
Galarza et al, 2021^22^	44	42.0	73%	NA	20.5%	6.8%	97.7%	NA	57.0
Guo et al, 2007^23^	11	36.7	45%	55%	18.2%	9.1%	81.8%	9.0%	39.6
Huang et al, 2022^24^	35	37.9	60%	63%	5.7%	2.9%	80.0%	0%	37.8
Jiang et al, 2017^18^	14	45.2	60%	22%	21.4%	21.4%	78.6%	21.4%	39.8
Medani et al, 2019^3^	36	51.0	86%	46%	30.6%	0%	36.1-80.6%^a^	11.1%	23.1
Murphy et al, 2011^25^	28	NA	NA	NA	NA	NA	63.0%	NA	NA
Murphy et al, 2016^26^	45	NA	NA	NA	NA	NA	93.3%	NA	NA
Neulen et al, 2011^7^	13	60.0	77%	77%	7.7%	7.7%	53.8%	7.7%	10.7
Potts et al, 2016^6^	35	51.9	83%	40%	31.4%	14.3%	46.5%	8.3%	8.0
Smith et al, 2011^27^	18	52.2	94%	11%	16.7%	5.6%	55.6%	0%	16.0
Sun et al, 2013^20^	38	41.4	63%	55%	13.2%	NA	97.4%	NA	21.0
Sun et al, 2013^19^	55	40.4	69%	53%	3.6%	3.6%	95.0%	NA	27.5
Tanaka et al, 2006^8^	12	50.6	42%	83%	16.7%	0%	83.3%	NA	31.7
Voyadzis et al, 2001^9^	10	48.1	80%	30%	20.0%	0%	70.0%	NA	31.7
Weigel et al, 2016^28^	13	51.8	69%	NA	7.7%	7.7%	29.2-54.2%^b^	15.4%	63.6
Xu et al, 2012^14^	13	37.8	40%	13%	7.7%	7.7%	84.6%	7.7%	40.1
Yucesoy et al, 2021^29^	40	28.4	88%	NA	27.5%	2.5%	82.5%	NA	96.0
Zheng et al, 2016^12^	22	42.6	91%	41%	27.3%	0%	81.8%	4.5%	20.5
**Current study**	**97**	**49.8**	**78%**	**38%**	**17.5%**	**11.3%**	**76.0%**	**12.4%**	**19.7**
Percutaneous									
Jiang et al, 2015^15^	42	34.3	52%	26%	16.7%	0%	85.7%	0%	24.0
Jiang et al, 2017^18^	56	45.2	60%	22%	12.5%	0%	100.0%	0%	39.8
Murphy et al, 2011^25^	100	54.0	84%	NA	8.0%	0%	48.5%	17.0%	7.3
Murphy et al, 2016^26^	213	NA	68%	48%	19.7%	0%	74.0%	16.9%	72.0
Endoscopic									
Wang et al, 2022^16^	15	41.3	60%	20%	0%	0%	80.0%	0%	15.6

The reoperation rate was defined as reoperations due to complications. NA = not applicable.

^a^According to MacNab criteria: excellent and good outcomes equal 36.1%, including fair outcomes, the improvement percentage increases to 80.6%.

^b^The outcome was derived from BNI and DNS scores. Depending on the cut-off, satisfactory outcomes were between 29-54%.

The results of the current study are summarized in Tables 2 and 3. Demographics and initial symptoms of the patients are presented in Table 2, and the radiological findings in Table 3 and in which comprehensive radiological measurements were available from 64 patients. Multiple cysts were more often found in females than in males in our cohort (48.7% vs 4.8%, *P* < .001).

**Table 2.** Patient Demographics, Symptoms, Complications, and Outcomes.

	All	Improvement	No Improvement	*P*-value
Patient number	97	73 (76.0%)	23 (24.0%)	-
Age (mean, range, years)	49.8 (24-73)	51.0 (24-73)	47.0 (28-70)	.125
Female	76 (78.4%)	54 (74.0%)	21 (91.3%)	.080
Male	21 (21.6%)	19 (26.0%)	2 (8.7%)	.080
Prior lower back surgery	12 (12.4%)	6 (8.2%)^C^	6 (26.1%)^C^	.034*
BMI^a^ (mean,±SD)	26.0 ± 5.1	26.1 ± 5.0	25.5 ± 5.9	.735
Symptoms				
Duration^b^ (mean, range, months)	31.7 (1.1-170.5)	30.6 (1.1-132.5)	36.3 (1.9-170.5)	.817
Motor				
Lower extremity weakness	30 (30.9%)	22 (30.1%)	7 (30.4%)	.978
Sensory (pain)				
Lower extremity (radicular)	86 (88.7%)	62 (84.9%)	23 (100%)	.061
Lower back and/or sacral	65 (67.0%)	54 (74.0%)	10 (43.5%)	.007*
Pelvis (perineal and genital)	13 (13.4%)	11 (15.1%)	2 (8.7%)	.727
Sensory (numbness or dysesthesia)				
Lower extremity	49 (50.5%)	34 (46.6%)	15 (65.2%)	.119
Pelvis (perineal and genital)	27 (27.8%)	18 (24.7%)	9 (39.1%)	.178
Bladder dysfunction	62 (63.9%)	49 (67.1%)	13 (56.5%)	.354
Bowel dysfunction	15 (15.5%)	13 (17.8%)	2 (8.7%)	.510
Complications				
Rate	17 (17.5%)	10 (13.7%)	7 (30.4%)	.113
Reoperation	11 (11.3%)	7 (9.6%)	4 (17.4%)	.452
Recurrence				
Rate	12 (12.4%)	6 (8.2%)	5 (21.7%)	.126
Reoperation	11 (11.3%)	5 (6.8%)	5 (21.7%)	.056

Percentages for “improvement” and “no improvement” represent the prevalence of each parameter within the subgroup.

^a^25 missing data points in BMI.

^b^22 missing data points in symptom duration.

**Table 3.** Radiological Findings and Outcomes.

	All	Improvement	No Improvement	*P*-value
Single cyst	59 (60.8%)	48 (65.8%)	10 (43.5%)	.057
Multiple cysts	38 (39.2%)	25 (34.2%)	13 (56.5%)	.057
Cyst size^a^				
Cyst diameter (cm)				
Mean, ± SD	3.4 ± 2.2	3.4 ± 2.2	3.6 ± 2.5	.932
Median	2.6	2.6	2.6	
Cyst volume (ml)				
Mean, ± SD	11.0 ± 16.5	10.7 ± 15.6	11.8 ± 19.2	.461
Median	3.3	3.9	2.6	
Bone erosion	76 (78.4%)	58 (79.5%)	17 (73.9%)	.575
Cysts origin^b^	114			
L5 nerve root	1 (1%)	-	-	-
S1 nerve root	24 (21%)	15 (20.5%)	5 (21.7%)	1.000
S2 nerve root	50 (44%)	29 (39.7%)	7 (30.4%)	.422
S3 nerve root	17 (15%)	8 (11.0%)	3 (13.0%)	.721
NOS^c^	22 (19%)	15 (20.5%)	2 (8.7%)	.346

Percentages for “improvement” and “no improvement” represent the prevalence of each parameter within the group.

^a^Comprehensive radiological measurements were available from 64 patients.

^b^Only single cyst origins evaluated in subcategories.

^c^Not specified.

All patients underwent surgical treatment performed by a single neurosurgeon. The patients were in a prone position, and the surgical site was identified with fluoroscopy. A partial sacral laminectomy was performed to expose the cyst. The cyst was dissected from the sacral bone and the adjacent structures and emptied with needle aspiration. Thereafter the cyst was wrapped with a suturable dural substitute (NeuroPatch, B. Braun SE, Germany). If the cyst could not be completely dissected from adjacent structures to allow wrapping, the cyst was resected and closed. In the case of a CSF leak, the closure was performed in a watertight manner with fibrin glue and absorbable patching (TachoSil, Takeda Pharmaceutical, Japan). No lumbar drainage was used by default. A total of 80 patients were treated with the wrapping technique and in the remaining 17 patients cyst resection was performed. The length of the hospital stay was 3.9 ± 3.0 days (mean ± SD).

Postoperative complications occurred in 17 (17.5%) patients. The most common complication was a CSF leak, which occurred in 10 patients. Two patients had a postoperative cauda equina syndrome and 2 patients had a superficial wound infection. Aseptic meningitis, persistent headache, and genital numbness were each reported once. Reoperations due to complications were performed in 11 patients, one of whom underwent three revisions due to a persistent CSF leak.

One patient was lost from follow-up and, accordingly, the outcomes of 96 patients were evaluated at the last follow-up. The length of the follow-up was 19.7 ± 32.9 months (mean ± SD, range 1.3 to 168.6 months). Thirty-six (37.5%) patients had an excellent outcome, 37 (38.5%) patients had a good outcome, 14 (14.6%) patients had a fair outcome, and 9 (9.4%) patients had a poor outcome. In total 76.0% of patients were classified to have an improvement in their symptoms (excellent and good outcome). The only parameters that differed between the 2 outcome groups were prior lower back surgery and the localization of pain in the lower back or sacral area. The 2 surgical techniques were compared in terms of outcome and no difference in the improvement rate was found between the wrapping group and the resection group (78.8% vs 62.5%, *P* = .201). Complication and reoperation rates did not differ statistically between the surgical techniques but cyst recurrence during postoperative follow-up was more common in the resection group (8.8% vs 29.4%, *P* = .034).

## Discussion

This is the second-largest study on the surgical outcome of Tarlov cysts ever published, reporting the outcome of 96 consecutive patients.^4^ In total, 76.0% of the patients experienced an improvement in their symptoms after surgery (excellent or good outcome). The outcomes were comparable with previous literature showing an unambiguous benefit of surgical treatment (Table 1).

Factors affecting the outcome of surgery are presented in Tables 1 and 2. No independent predictors of outcome were found in the regression analysis. Patient demographics did not differ between the outcome groups but there were differences in the preoperative symptomatology. The improvement rate was significantly higher in patients who had lower back or sacral pain as their initial symptom as compared to those who did not (74.0% vs 43.5%, *P* = .007). Radicular pain, on the other hand, was present in all patients who did not improve after surgery and in 84.9% who did (*P* = .061). Thus, based on the current results, radicular pain as a sole symptom seems to be an insufficient indication to operate on Tarlov cysts. The local pain, on the other hand, seems to respond to treatment. It is likely caused by the actual pressure of the cyst on the surrounding tissues, and it has been correlated with bony erosion,^10^ which in turn, has been hypothesized to be associated with the compression of periosteal pain fibers.^7^ It has been, self-evidently, suggested that bony erosion is more common in larger cysts.^10^ However, in the current study, cyst size and the presence of bone erosion did not differ between the outcome groups. Thus, the mechanisms of local pain can not only be attributed to the radiological parameters and other factors, eg inflammatory mechanisms, are likely to contribute to the pain. Therefore, the morphometry of the cyst and sacral bone erosion should not be used as the basis to predict the outcome, but the symptomatology should play a crucial role in the decision-making.

In previous literature, cyst size has been speculated to affect the outcome, and patients with cysts larger than 1-1.5 cm regarded to benefit from surgery.^7,9^ However, a correlation between cyst size and the outcome has not been established in larger study populations.^4,21^ Also in the current study, cyst characteristics did not affect the outcome. It is notable that the mean and median size of cysts was well above the previously stated limit, which is by no means in conflict with the prevailing consensus that small cysts are likely to be incidental and should not be operated on. In the population of our study, the number of cysts was close to reaching statistical significance (*P* = .057) with multiple cysts more common among patients who did not benefit from surgery and a single cyst found more often in patients who experienced an improvement. The presence of multiple cysts has also previously been speculated to lead to a poorer outcome,^7^ which is likely due to more limited treatment options and complex techniques.

Our study and the literature review, as shown in Table 1, are conclusive on a sex-related predisposition of females to develop symptomatic Tarlov cysts. All these studies are operative series, and it may be postulated that females are more likely to seek operative treatment, but the sex-related difference was also seen in a radiological meta-analysis by Klepinowski, and colleagues from 2021^5^ confirming the finding. There were no statistical differences in the outcomes based on sex but the percentage of males was somewhat higher in the improvement group as compared to the no-improvement group (26.0% vs 8.7%, *P* = .080). Furthermore, multiple cysts were more often found in females (*P* < .001).

The literature describes an abundance of treatment options for Tarlov cysts ranging from minimally invasive aspiration techniques to endoscopy and open surgery, all of which have their own nuances, and even surgical treatment comprises multiple different operation techniques. Surprisingly, all of them seem to produce relatively comparable results (Table 1) and a comparison of different techniques has not shown clear superiority of 1 over another.^30^ Accordingly, in the current study, no differences were found between the 2 applied operation techniques. Regardless of the treatment technique, it has been postulated that the treatment should be prompt if a symptomatic Tarlov cyst is suspected as the background of symptoms after an adequate diagnostic workup.^21^ In the current study, the symptom duration did not differ between the outcome groups but we found that patients who did not achieve an improvement in their symptoms had previously undergone lower back surgery more often (26.1% vs 8.2%, *P* = .034). Thus, based on the current results, symptom duration cannot be used as a predictor of the outcome but the higher prevalence of previously surgically treated spinal co-morbidities in patients with an unsatisfactory outcome may actually have been an indicator of an asymptomatic cyst in the first place.

In addition to patient- and cyst-related parameters to predict the outcome of surgery, a diagnostic workup algorithm has been suggested by Fletcher et al^2^ including CT myelography and prognostic cyst aspiration. CT myelography shows, whether the cyst communicates freely with the CSF space and in non-communicating cysts aspiration may be used to simulate the effect of surgery and to help to identify a population of patients, who are likely to gain a benefit from surgery. This approach is also currently applied in our institution since it could provide a potential outcome predictor in future studies.

This is a large but retrospective study of an unselected patient population of consecutive patients over a relatively long time-span. Due to the nature of the study, no detailed patient-reported outcome data were available. The imaging protocol was not uniform and postoperative imaging was not performed if the recovery was uneventful and the outcome was good. Also, the clinical follow-up in these patients was not prolonged beyond a single routine postoperative outpatient clinic visit. However, the rarity and somewhat controversial nature of the condition limit the applicability of large-scale prospective or randomized studies, and therefore retrospective registers remain the main research strategy.

In conclusion, operative treatment of Tarlov cysts in a selected patient population will likely produce alleviation in the symptoms when balanced with the complication rate and profile of surgery. The findings of the current study can be applied as an aid in the decision-making process for selecting candidates for surgery. Preoperative lower back or sacral pain is a potential indicator for improvement after surgery. However, it is not possible to provide clear-cut indications for surgical treatment of Tarlov cysts, since the regression analysis did not find any single symptom, demographic characteristic or radiological parameter to independently predict the outcome. In future studies the quality of life of patients with Tarlov cysts should be addressed and the results of surgery should be compared with the natural course and long-term outcome of untreated cysts.
